# Decoding Steady-State Visual Evoked Potentials From Electrocorticography

**DOI:** 10.3389/fninf.2018.00065

**Published:** 2018-09-26

**Authors:** Benjamin Wittevrongel, Elvira Khachatryan, Mansoureh Fahimi Hnazaee, Flavio Camarrone, Evelien Carrette, Leen De Taeye, Alfred Meurs, Paul Boon, Dirk Van Roost, Marc M. Van Hulle

**Affiliations:** ^1^Laboratory for Neuro- and Psychophysiology, Department of Neurosciences, KU Leuven, Leuven, Belgium; ^2^Laboratory of Clinical and Experimental Neurophysiology, Neurology Department, Ghent University Hospital, Ghent, Belgium; ^3^Department of Neurosurgery, Ghent University Hospital, Ghent, Belgium

**Keywords:** BCI, ECoG, scalp-EEG, SSVEP, decoding, beamforming, CCA, cortex

## Abstract

We report on a unique electrocorticography (ECoG) experiment in which Steady-State Visual Evoked Potentials (SSVEPs) to frequency- and phase-tagged stimuli were recorded from a large subdural grid covering the entire right occipital cortex of a human subject. The paradigm is popular in EEG-based Brain Computer Interfacing where selectable targets are encoded by different frequency- and/or phase-tagged stimuli. We compare the performance of two state-of-the-art SSVEP decoders on both ECoG- and scalp-recorded EEG signals, and show that ECoG-based decoding is more accurate for very short stimulation lengths (i.e., less than 1 s). Furthermore, whereas the accuracy of scalp-EEG decoding benefits from a multi-electrode approach, to address interfering EEG responses and noise, ECoG decoding enjoys only a marginal improvement as even a single electrode, placed over the posterior part of the primary visual cortex, seems to suffice. This study shows, for the first time, that EEG-based SSVEP decoders can in principle be applied to ECoG, and can be expected to yield faster decoding speeds using less electrodes.

## 1. Introduction

Electrocorticography (ECoG) records electrophysiological signals from the cortical surface, without the electrodes penetrating the cortical tissue, unlike multi-electrode arrays and deep brain electrodes. This recording modality has led to new opportunities for Brain-Computer Interfacing (BCI). While scalp-EEG avoids surgical interventions, it comes with inferior signal quality and spatial resolution, due to volume conduction, which challenges the speed/accuracy ratio when judging decoding performance (Schalk, 2010; Schalk and Leuthardt, 2011). Additionally, success in EEG-based BCI applications depends on alleviating eye and head motion artifacts (Croft and Barry, 2000), including those causing minute electrode displacements, the drying out of the conductive gel that ensures low impedance contact with the subject's scalp, and the time-consuming electrode set-up and the ensuing retraining of the BCI prior to each use (for review, see Lacko et al., 2017). When relying on micro-electrode arrays such as the Utah array (Hochberg et al., 2006; Collinger et al., 2013) one is facing permanent damage to the cortical tissue and its vasculature leading to histological processes and fibrous scar tissue formation (Schalk and Leuthardt, 2011) and brain plasticity processes that deteriorate the signal quality and whence BCI performance (Shain et al., 2003). Compared to scalp-recorded EEG, ECoG signals are less contaminated by eye and motion artifacts (Ball et al., 2009), have higher amplitudes (Schalk and Leuthardt, 2011), a broader bandwidth (Staba et al., 2002), and higher spatial resolution (Miller et al., 2009b). Several studies were able to discriminate fine real (i.e., performed) movements from ECoG recordings such as hand extension and flexion (Jiang et al., 2017), hand movement direction (Leuthardt et al., 2004) and individual finger movements (Miller et al., 2009a), all of which are not be feasible from EEG recordings. ECoG-based BCI have furthermore shown great promise for long-term recording sessions (Chao et al., 2010; Wang et al., 2013) with reports of 262 days BCI-use without signal degradation (Vansteensel et al., 2016).

While motor paradigms are raising great hopes to eventually achieve intuitive control over prosthetic limbs, currently, instead of continuous, only discrete control can be achieved (i.e., discrimination between distinct imagined motor tasks) (Onose et al., 2012; Vansteensel et al., 2016) as the extraction of reliable limb trajectory information is still largely beyond reach (Korik et al., 2018). In the discrete setting, visual paradigms vastly outperform sensorimotor paradigms in terms of accuracy, number of selectable targets and information transfer rate (ITR) (Nicolas-Alonso and Gomez-Gil, 2012). Several studies have investigated the feasibility of ECoG for visual BCI paradigms, such as the P300 Event-Related Potential (Brunner et al., 2011; Krusienski and Shih, 2011; Speier et al., 2013) and the code-modulated Visual Evoked potential (Kapeller et al., 2013). The implanted electrodes were mainly located over frontal, temporal, parietal and occipital areas, albeit not reaching to the primary visual cortex (V1) and merely a few electrodes to the secondary visual cortex (V2) (Speier et al., 2013). However, to the best of our knowledge, the Steady-State Visual Evoked Potential (SSVEP), the most performant visual BCI paradigm (Chen et al., 2015b), has not yet been assessed in terms of ECoG (or even intracranially recorded signals in general). Subdural electrode implants primordially serve a clinical purpose such as to localize epileptogenic foci of drug-resistant epilepsy and the mapping of eloquent cortex prior to resective surgery. Of all epilepsy cases, only 5–10% have an occipital lobe origin (Sveinbjornsdottir and Duncan, 1993). Foci in the first visual layers (V1, V2) are, moreover, considered inoperable, as resection would leave the patient blinded, thereby normally not warranting electrode implantation. As, in general, only intractable cases of epilepsy are considered for implantation and given the above-mentioned precautions, patients with ECoG grids over primary visual areas are extremely rare. Furthermore, as the SSVEP paradigm relies on periodically flickering stimuli, photo-sensitive epilepsy patients are excluded from participation in an SSVEP study, further limiting the opportunities to study SSVEP responses over (primary) visual areas in human subjects. There are very few SSVEP-based studies with implanted electrodes in the occipital cortex, which are all limited to electrode strips (Kamp et al., 1960; Krolak-Salmon et al., 2003; Winawer et al., 2013), thereby not providing a wider view of the cortical activations, and none of these studies investigated SSVEP decoding performance. We report on a single case study where a large subdural grid was covering the entire right occipital cortex of a human subject.

When used in BCI, SSVEP-selectable targets are traditionally encoded with different flickering frequencies (Regan, 1979; Middendorf et al., 2000) or phases (Lee et al., 2010; Lopez-Gordo et al., 2010; Manyakov et al., 2012), but in order to maximize the number of selectable targets, a joint frequency-phase encoding has been suggested (Jia et al., 2011). Many SSVEP decoding algorithms have been described (for overviews, see Liu et al., 2013; Wittevrongel and Van Hulle, 2017; Lotte et al., 2018), but the more advanced ones are based on adapted versions of canonical correlation analysis (CCA) (Lin et al., 2006; Bin et al., 2009; Pan et al., 2011; Zhang et al., 2011, 2014; Chen et al., 2014; Nakanishi et al., 2014; Yin et al., 2015; Abu-Alqumsan and Peer, 2016; Vu et al., 2016), with the filterbank CCA (Chen et al., 2015a) yielding the highest decoding accuracy, and on a spatiotemporal extension of the beamforming algorithm (Wittevrongel and Van Hulle, 2017), successfully used for SSVEP (Wittevrongel and Van Hulle, 2016c) as well as several other visual BCI paradigms (Wittevrongel and Van Hulle, 2016a,b; Wittevrongel et al., 2017). While both filterbank CCA (fbCCA) and spatiotemporal beamformer (stBF) yield state-of-the-art decoding performance on scalp-recorded EEG, it is unclear how well they perform on ECoG as the signal properties are different (Buzsáki et al., 2012; Ritaccio et al., 2015). This study aims to investigate SSVEP decoding from the cortical surface and to compare the obtained accuracies to the ones from traditional scalp-recorded signals.

## 2. Methods

### 2.1. Participants

A 38-year old male patient (right-handed) with refractory, non-photosensitive epilepsy participated in the study. He was admitted to the hospital (UZ Gent) for monitoring seizure activity and functional mapping of eloquent cortex (visual, language, etc.). A subdural grid of 48 platinum electrodes embedded in silastic (Ad-Tech, USA) was implanted over the right occipital cortex. Additionally, a control group of eight healthy subjects (5 male, mean age 24.5 years, ranging from 19 to 30 years, 1 left-handed) were recruited for scalp-EEG recording. Previous studies have shown that BCI accuracy is not affected by age (Allison et al., 2010), thereby not requiring age-matched controls. All recruited participants, including the patient, had normal or corrected-to-normal vision and were informed, prior to participating, about the aim of the study, the experimental procedure, and what would be done with the recorded data, after which they signed the informed consent form. The study was conducted according to the latest version of the Declaration of Helsinki (2013) following prior approval from the ethical committee of Ghent University Hospital (ECoG study) and Leuven University Hospital (UZ Leuven) (EEG study).

### 2.2. Experimental procedure

The experimental interface consisted of six identical rectangular targets (8.8 × 5.8 cm) presented on a 60 Hz LCD monitor. A number (from 1 to 6) was displayed in the center of each target, serving as a fixation point for the corresponding rectangle. The targets adopted a green color to reduce the visual demand (Cao et al., 2012; Tello et al., 2015) and the risk of inducing an epileptic seizure (Kaiser, 1984). All subjects were seated approximately 60 cm from the screen. At this distance, the visual angle spanned by the rectangles was 8.4° × 5.5°, and the distance from the fixation point to the edge of the neighboring target was 5.5° horizontally and 4.1° vertically. The fixation numbers spanned a visual angle of approximately 0.35° × 0.70°.

**Figure 1:**
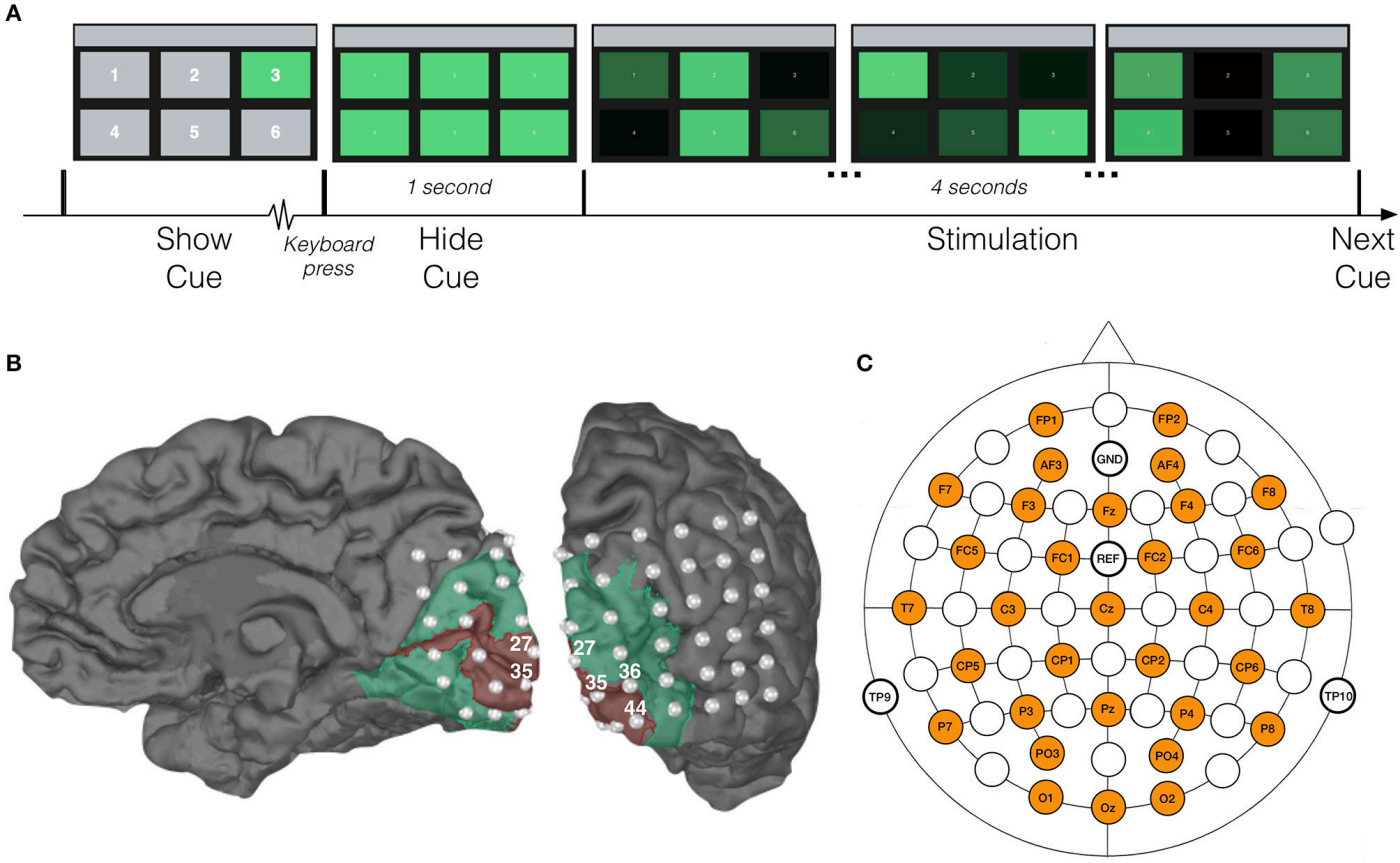
Experimental setup. **(A)** Time-course of one trial with characteristic displays. For the sake of exposition, target numbers in the first display are larger than those shown to our subjects. **(B)** Visualization of the patient's right hemisphere with location of implanted ECoG electrodes. Red and green areas correspond to V1 and V2, respectively, and were extracted with Freesurfer (Fischl, 2012). **(C)** Location of scalp-EEG electrodes for the control experiment.

In order to investigate the decoding performance for different stimulus settings, the experiment consisted of four sessions, in each of which all rectangles were assigned a unique combination of frequency and phase (Table 1). Each session consisted of 90 trials. At the beginning of each trial, one of the targets was cued by maintaining its green color while the other rectangles were shown in gray (Figure 1A). The subject was asked to direct his/her gaze at the cued target and maintain focus. When the subject pressed a key on the keyboard, all rectangles regained their green color, and 1 s later, the 4-s stimulation was initiated, during which all targets were flickering in accordance with their frequency-phase combination, achieved by sinusoidally modulating their luminosities (Manyakov et al., 2013). Between trials, the subject was allowed to take a short break, and a longer one (around 5 min) between sessions. In each session, all targets were cued 15 times in pseudorandom order. The experiment was implemented in Matlab, using the Psychophysics Toolbox extensions (Brainard and Vision, 1997; Pelli, 1997; Kleiner et al., 2007).

**Table 1:** Target frequency-phase combinations for each session, represented as [frequency (Hz)/phase (radians)].

**Session**	**Target**					
	**1**	**2**	**3**	**4**	**5**	**6**
1	12/0	14/$\frac{2\pi }{3}$	12/$\frac{4\pi }{3}$	14/$\frac{4\pi }{3}$	12/$\frac{2\pi }{3}$	14/0
2	13/0	14/$\frac{2\pi }{3}$	13/$\frac{4\pi }{3}$	14/$\frac{4\pi }{3}$	13/$\frac{2\pi }{3}$	14/0
3	11/0	15/π	13/0	13/π	11/π	15/0
4	13/0	15/π	14/0	14/π	13/π	15/0

### 2.3. Scalp-EEG and ECoG acquisition

For the patient, 48-channel ECoG and 27-channel scalp-EEG (following the international 10-20 system) data were recorded simultaneously at a sampling rate of 1,024 Hz using an SD LTM 64 Express (Micromed, Italy) medically certified device. Each ECoG electrode had a 4.0 mm diameter with 2.3 mm exposure and 10 mm inter-contact spacing. The subdural grid (6 × 8 contacts) was covering the right occipital cortex: convexity and mesial inter-hemispheric cortex (Figure 1B). Functional mapping confirmed that a large part of the grid was located over the primary and associative visual cortex. Due to insufficient quality of the simultaneously recorded scalp-EEG (dried conductive gel, as well as the influence of scarred and swollen tissue), the patient's scalp-EEG was excluded from further analysis.

The scalp recorded EEGs from the control subjects were acquired with a Synamps RT device (Neuroscan, Australia), using 32 active Ag/AgCl electrodes evenly distributed over the scalp (Figure 1C) and at a sampling rate of 2 kHz. The electrodes were placed using an electrode cap (EasyCap GmbH, Germany) with fixed holes according to the international 10/20 system. Conductive gel was applied at each electrode, and the impedance between skin and electrodes was kept below 2 kΩ. As SSVEPs are less susceptible to ocular artifacts (i.e., blinks and saccades) (Regan, 1989; Vialatte et al., 2010), no electro-oculogram (EOG) was recorded and no artifact correction was applied in this study.

### 2.4. Localization of ECoG electrodes

Based on the pre-implantation MRI scan of the patient, cortical reconstruction, and volumetric segmentation was performed with the Freesurfer image analysis suite (version 5.3.0) (Fischl, 2012). The Freesurfer output was then loaded into Brainstorm (Tadel et al., 2011) and co-registered with the post-implant CT. The coordinates of the implanted electrodes were then obtained by visual inspection and mapped onto the subject's cortical surface.

Figure 1B shows the locations of the implanted electrodes, with indication of the primary (V1) and associative (V2) visual cortices.

### 2.5. Processing

The processing of the ECoG and EEG data differs only in the used re-referencing. The raw ECoG data was offline re-referenced to the grid's average (Common Average Reference, CAR), while the EEG data was offline re-referenced to the average of both mastoid electrodes (TP9 & TP10).

Since each classifier (see next section for their description) requires different filtering ranges, all recordings were stored four times, but with different filtering ranges: a fourth-order Butterworth filter between 0.5 and 40 Hz for the naive classifier, between 4 and 20 Hz for the spatiotemporal beamformer (Wittevrongel and Van Hulle, 2016b), and between 8 and 70 Hz and between 16 and 70 Hz for the filterbank CCA algorithm (Chen et al., 2015a), respectively. The filtered EEG and ECoG recordings were then cut into 4-s trials time-locked to the onset of the stimulation, downsampled to 500 Hz, labeled with the corresponding cue, and stored for further analysis.

### 2.6. Classifiers

In this study, we assessed the performance of two state-of-the-art classifiers, filterbank CCA (fbCCA) and spatiotemporal beamformer (stBF), and a simple (called naive) classifier based on the Fourier transform in combination with a phase estimation method.

#### 2.6.1. Naive classifier

This classifier is the most straightforward one to identify gazed targets. First, the most prominent frequency (among the used stimulation frequencies) in a trial was determined from the signal's power spectral density (PSD), obtained after applying the Fourier transform. Then, this frequency was used to estimate the phase of the trial. Finally, the predicted target was taken as the one whose frequency and phase best corresponded to the extracted frequency and phase. This is now explained in more detail.

Training the naive classifier involved the estimation of the phase Φ_*i*_ of each target *i*∈[1..6], obtained by applying the following equation to each training trial *s* of target *i* and averaging their values (Walker, 1988; Jia et al., 2011; Manyakov et al., 2013):

(1) $$\begin{array}{l} \varphi =\text{Arg}\left(F_{s}(f_{i})\right)\\ \;=\text{Arg}\left(\sum_{t}s(t)\text{ cos}(2\pi f_{i}t)+j\sum_{t}s(t)\text{ sin}(2\pi f_{i}t)\right)\\ \;=\text{atan2}\left(\sum_{t}s(t)\text{ cos}(2\pi f_{i}t),\sum_{t}s(t)\text{ sin}(2\pi f_{i}t)\right), \end{array}$$

with *t* the sample in time units, *f*_*i*_ the frequency of target *i*, $F_{s}$ the discrete Fourier transform of *s*(*t*), and $j=\sqrt{-1}$.

To classify a trial, the PSD was calculated using the Fourier transform with a Hamming window whose size was equal to the length of the trial. For each of the stimulation frequencies *f*_*i*_, the power was calculated as the average in a narrow (0.5 Hz) band, centered at frequency *f*_*i*_:

(2) $$\begin{array}{r} p_{f_{i}}=\overset{f_{i}+0.25}{\underset{f_{i}-0.25}{\int }}PSD\left(x\right)\text{d}x, \end{array}$$

where *x* is the range of the PSD spectrum. The predicted frequency $\hat{f}$ was given by *f*_*i*_ with the highest power, and further used for estimating the phase $\hat{\varphi }$ of the trial, using Equation 1. Finally, the phase difference δ*i* between Φ_*i*_ and $\hat{\varphi }$ was calculated for all targets *i* that were encoded by frequency $\hat{f}$, using the *CircStat* toolbox (Berens et al., 2009). The gazed target was predicted as the one with the closest-to-zero phase difference.

#### 2.6.2. Filterbank CCA

The CCA algorithm was originally used in the context of SSVEP-BCI for the identification of frequency-encoded SSVEP targets (Lin et al., 2006). The algorithm finds linear combinations ${\text{w}}_{x}\in {\mathbb{R}}^{1\times m}$ and ${\text{w}}_{y}\in {\mathbb{R}}^{1\times k}$ for multivariate sets **X** ∈ ℝ^*n*×*m*^ and **Y** ∈ ℝ^*n*×*k*^, such that their linear combinations $\text{x}=\text{X}{\text{w}}_{x}^{⊺}$ and $\text{y}=\text{Y}{\text{w}}_{y}^{⊺}$ are maximally correlated (Härdle and Simar, 2015).

In the context of SSVEP, **X** is the measured (multi-channel) EEG and **Y**_*i*_ is a set of sine and cosine signals with the stimulation frequency of target *i* and a number of harmonics (*h* = *k*/2).

(3) $$Y_{i}=\left(\begin{array}{c} \sin(2\pi f_{i}t)\\ \cos(2\pi f_{i}t)\\ \vdots \\ \sin(2\pi hf_{i}t)\\ \cos(2\pi hf_{i}t) \end{array}\right)$$

Since the original algorithm does not contain any phase information, an extension was proposed (Nakanishi et al., 2014) to discriminate joint frequency-phase encoded SSVEP targets by incorporating individual calibration data. The filterbank CCA method (Chen et al., 2015a) combines the aforementioned CCA extension (Nakanishi et al., 2014) with a filterbank approach (Vetterli and Herley, 1992). By analyzing the signals in different sub-bands, information originating from the fundamental and harmonic components are examined more efficiently and aggregated at a later stage. In this study, the number of harmonics *h* for the predefined signals **Y**_*i*_ was set to 3 and the sub-bands used were [8-70] Hz and [16-70] Hz as in (Chen et al., 2015b).

#### 2.6.3. Spatiotemporal beamforming

The spatiotemporal extension (van Vliet et al., 2015) (termed here stBF) of the original beamforming algorithm (Van Veen and Buckley, 1988) has first been proposed for the analysis of the N400 ERP in the context of semantic processing, and since then applied in the context of BCIs (Wittevrongel and Van Hulle, 2017) for target identification based on the P300 ERP (Wittevrongel and Van Hulle, 2016a), SSVEP (Wittevrongel and Van Hulle, 2016b,c) and cVEP (Wittevrongel et al., 2017) paradigms.

Spatiotemporal beamforming estimates the contribution of a predefined spatiotemporal pattern to new data. Each trial is cut into consecutive, non-overlapping segments whose length is equal to the period of the expected frequency (Luo and Sullivan, 2010; Manyakov et al., 2010). If the frequency is present in the signal, the averaged segments should resemble a sine-wave of a single period. If not, averaging the segments will cancel out any patterns and result in a “flat” response. As the segments are time-locked, the averaged pattern also accounts for the phase of target stimulation.

Since each target elicits a different brain response (cf., the unique frequency/phase combinations), each target requires a unique activation pattern, hence training the classifier involves calculating six spatiotemporal beamformers, one for each target.

The *activation patterns* and the beamformers were calculated from the training data as follows: let ${\text{T}}_{i}\in {\mathbb{R}}^{m\times t\times r}$ be the trials obtained during the training session in response to the cued target *i*∈[1..6], where *m* represents the number of channels, *t* the number of samples and *r* the number of trials, and *f*_*i*_ be the stimulation frequency of target *i*. Applying the time-domain approach to all trials in **T**_*i*_ with *f*_*i*_ the returned segments ${\text{S}}_{i}\in {\mathbb{R}}^{m\times n\times k}$, where *n* is the number of samples corresponding to one period of frequency *f*_*i*_ and *k* the total amount of segments obtained from all trials in **T**_*i*_. The spatiotemporal activation pattern ${\text{A}}_{i}\in {\mathbb{R}}^{m\times n}$ for target *i* can now be obtained as the average of all *k* segments.

The *spatiotemporal beamformer* was calculated as a linearly-constrained minimum-variance (LCMV) beamformer ${\text{w}}_{i}\in {\mathbb{R}}^{\left(mn\right)\times 1}$ for target *i* as follows: let ${\text{E}}_{i}\in {\mathbb{R}}^{\left(mn\right)\times k}$ be the matrix where each row was obtained by concatenating the rows of a corresponding segment *S*_*i*_[^*^, ^*^, *k*], $\Sigma_{i}\in {\mathbb{R}}^{\left(mn\right)\times \left(mn\right)}$ the covariance matrix of **E**_*i*_, and **a**^⊺^ ∈ ℝ^1×(*mn*)^ a vector containing the concatenated rows of **A**_*i*_. The LCMV beamformer under constraint ${\text{a}}_{i}^{⊺}{\text{w}}_{i}=1$ can now be calculated using the method of Langrage multipliers (Van Veen et al., 1997):

(4) $$\begin{array}{r} w_{i}=\frac{\Sigma_{i}^{-1}a_{i}}{a_{i}^{⊺}\Sigma_{i}^{-1}a_{i}}, \end{array}$$

and applied to the data as a simple weighted sum: *y* = **sw**_*i*_, where **s** ∈ ℝ^1×(*mn*)^ indicates the concatenated rows of an input segment ${\text{S}}_{in}\in {\mathbb{R}}^{m\times n}$.

The feature vector for a trial **T** was given by the beamformer outputs *y*_*i*_ for each target *i*. To obtain *y*_*i*_, the trial was cut into segments of length one period of frequency *f*_*i*_, the segments were averaged and filtered using the corresponding beamformer **w**_*i*_.

Based on the feature vector, a prediction was made of the target gazed by the subject. As the feature vectors contain estimates of the degree to which the activation pattern of each target is present in the trial, the target having the highest score was marked as winner [i.e., prediction = max(*y*_*i*_)].

Note that longer trials can be cut into more segments, leading to more reliable average segment responses that can be input to the beamformers. Prior to training and classification, trials were bandpass filtered between 4 and 20 Hz.

### 2.7. Performance evaluation

The decoding accuracy of all classifiers was estimated offline using a stratified 5-fold cross-validation strategy. We estimated performance for varying signal lengths, from 250 ms to 4 s in steps of 250 ms. Note that only the testing trials were shortened, while the training trials remained 4 s long. Single-electrode performance was assessed for all three classifiers, while multi-electrode performance was only estimated for the two state-of-the-art classifiers (filterbank CCA and spatiotemporal beamformer).

### 2.8. Statistical estimation of the patient's scalp-EEG performance

As the patient's scalp-EEG was not of sufficient quality for use in our analysis, we statistically estimated his scalp-EEG accuracy for the single-electrode case using an imputation analysis based on phase variability. For each trial, the phase was calculated using Equation 1. Albeit that the measured phase on the scalp might be different from the cortical phase measurement due to the mixing of different cortical activities, this should not affect its trial-to-trial variability. First, we verified that the phase variability (measured as the circular standard deviation; Berens et al., 2009) of the ECoG signal (at electrode 36) is not significantly different from the scalp-recorded phase variability at channel Oz of our control subjects. To this end, the Kuiper's test was used (*CircStat* toolbox; Berens et al., 2009). Next, for each session, an imputation analysis with 100 iterations was performed in which the patients scalp-EEG decoding accuracy was treated as missing value. The patient's trial-to-trial phase variability of the session under consideration (at electrode 36) was used, together with the phase variabilities and decoding accuracies of all sessions from the control group to estimate the patient's scalp-EEG decoding accuracy.

### 2.9. Electrode selection

While many approaches have been described to recruit electrodes for multi-electrode analysis (Lal et al., 2004; Schröder et al., 2005; Lv and Liu, 2008; Arvaneh et al., 2011; Barachant and Bonnet, 2011), we used greedy forward selection: we iteratively added the electrode that resulted in the largest accuracy increase until no further improvement could be achieved or 100% accuracy was reached. As an optimization criterion, we used the average classification accuracy across stimulation lengths (from 250 ms to 4 s in steps of 250 ms, see above). This procedure was repeated for each classifier separately, in order to maximize their individual accuracies.

While the greedy approach does not guarantee optimal performance (as it does not backtrack on the choices made), it is universal, simple and straightforward to implement compared to other selection strategies. As the main aim of this study was to compare ECoG- and EEG-based SSVEP target decoding accuracy, the selection of the optimal electrode set was not deemed important in this study. Furthermore, the greedy selection approach takes into account the differences inherent to the classifiers (e.g., fbCCA solely aims at detecting the frequency-phase combinations while stBF estimates the contribution of a given activation pattern and takes into account information about noise sources and non-targeted patterns), and is often described as a *wrapper* approach (Guyon and Elisseeff, 2003).

### 2.10. Statistics

For the EEG subjects, the significance of the differences in classifier accuracies was assessed with the (two-tailed) Wilcoxon signed rank test. We adopted this non-parametric test since the distributions did not consistently follow a Gaussian distribution. The significance threshold was set to 0.05 for the multi-electrode case, and was corrected to 0.0167 ($=\frac{0.05}{3}$) for the single-electrode case, using the Bonferroni correction for multiple comparisons.

## 3. Results

Our subjects were shown a visual interface constituting of six rectangular targets, each one periodically flickering at a unique frequency and phase (Table 1). All subjects performed four sessions of 90 trials. In each trial, the subject was asked to direct his/her gaze to a cued target and to maintain focus during the ensuing 4-s flickering stimulation (Figure 1). In each session, all targets were cued 15 times in pseudorandom order.

### 3.1. Single-electrode performance

As this is the first study on ECoG-based SSVEP decoding, we started with an exploratory analysis of target detection accuracy of individual electrodes when using fbCCA (Chen et al., 2015a) and stBF (Wittevrongel and Van Hulle, 2016b), and a naive classifier based on spectral analysis but extended with phase modeling. The naive classifier has been applied to scalp-EEG recordings of SSVEP responses (for review, see Liu et al., 2013), but now considered outdated in terms of accuracy, hence, it is interesting to see to what extend that classifier could benefit from the improved ECoG signal quality.

**Figure 2:**
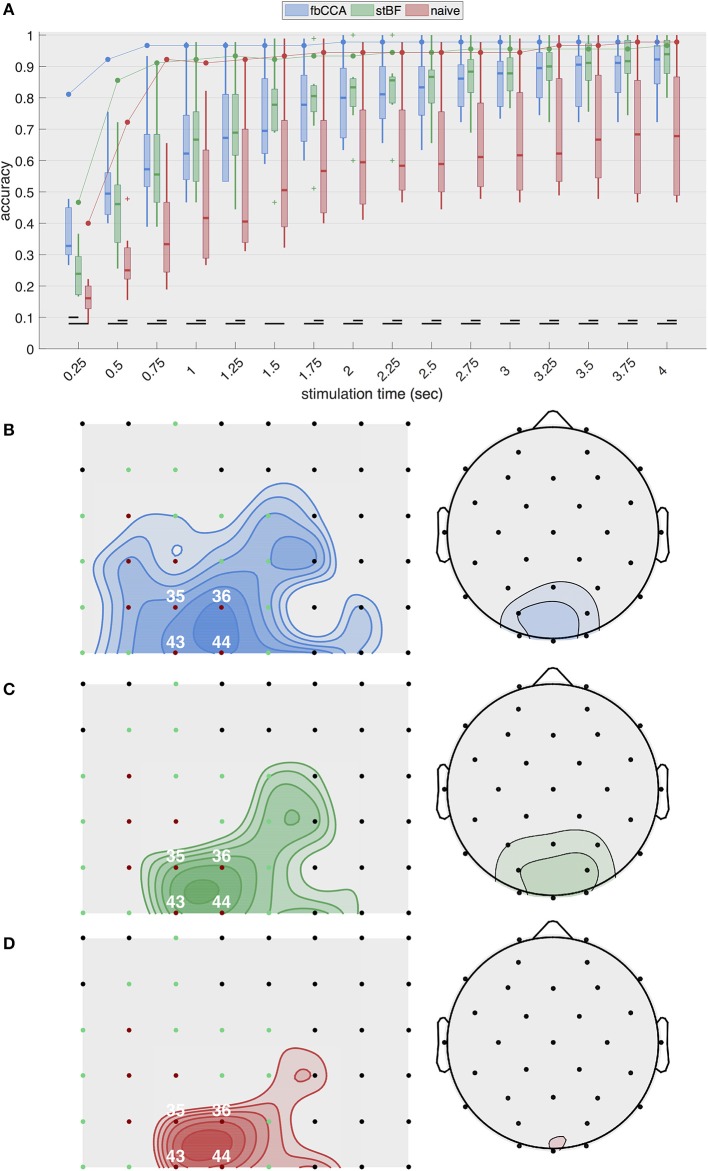
Single-electrode performance. **(A)** Best single-electrode accuracies obtained with the three classifiers plotted as a function of stimulation lengths during the second session. The full line indicates accuracies based on cortical recordings and the boxplots summarize accuracies based on scalp-EEG (control subjects). **(B-D)** Overview of accuracies (averaged across all stimulation lengths) for all cortical electrodes (left) and scalp channels (right) for **(B)** fbCCA, **(C)** stBF, and **(D)** naive classifier. Subdural electrodes indicated in red and green indicate V1 and V2, respectively. Iso-accuracy lines on the subdural grids (left panels) start at 75% accuracy and increase in steps of 5% and on the scalp plots (right panels) start at 50% accuracy and increase in steps of 10%.

Figure 2A shows the highest single-electrode decoding accuracies obtained from the three classifiers for increasing stimulation lengths during the second session. The results of the other sessions are equivalent and are shown in Figures S1–S3 in the Supplementary Material. For ECoG, the two state-of-the-art classifiers, as well as the naive classifier, exhibit a rapid increase in accuracy with increasing stimulation lengths up to 0.75 s of stimulation, after which the accuracy only improves marginally or remains constant. fbCCA is the most accurate classifier with an accuracy of 96.7% for 0.75-s stimulation, compared to 91.1 and 92.2% for stBF and the naive classifier, respectively. Using long stimulation (i.e., 4 s), the accuracy converges to 97.8% for the fbCCA and naive classifiers, and 96.7% for stBF. For scalp-EEG, the accuracy at the best electrode of each subject steadily increases with increasing signal lengths, but remains considerably lower than the ECoG-based accuracy (e.g., 79.6±13.5% for EEG compared to 97.8% for ECoG with fbCCA at a stimulation length of 2 s). Here, the accuracy of the naive classifier is consistently lower than that of the state-of-the-art classifiers, and is significantly different for all stimulation lengths above 0.25 s. At least 1.5 s of stimulation is needed for scalp-EEG to surpass the 70% accuracy threshold deemed necessary to establish reliable communication (Kübler et al., 2004; Kübler and Birbaumer, 2008; Brunner et al., 2011; Combaz et al., 2013). In comparison, using ECoG, all classifiers surpass this threshold within 0.5 s of stimulation.

Figures 2B–D summarize the average decoding accuracy across all stimulation lengths for each individual electrode during the second session. The EEG electrodes over the middle occipital area (electrode Oz) yield the highest accuracies, as expected. The subdural electrodes located over the posterior part of the primary visual cortex (electrodes 36 and 44) most accurately decode the intended target. These results are consistent across classifiers and sessions (see Supplementary Material for the other sessions).

**Figure 3:**
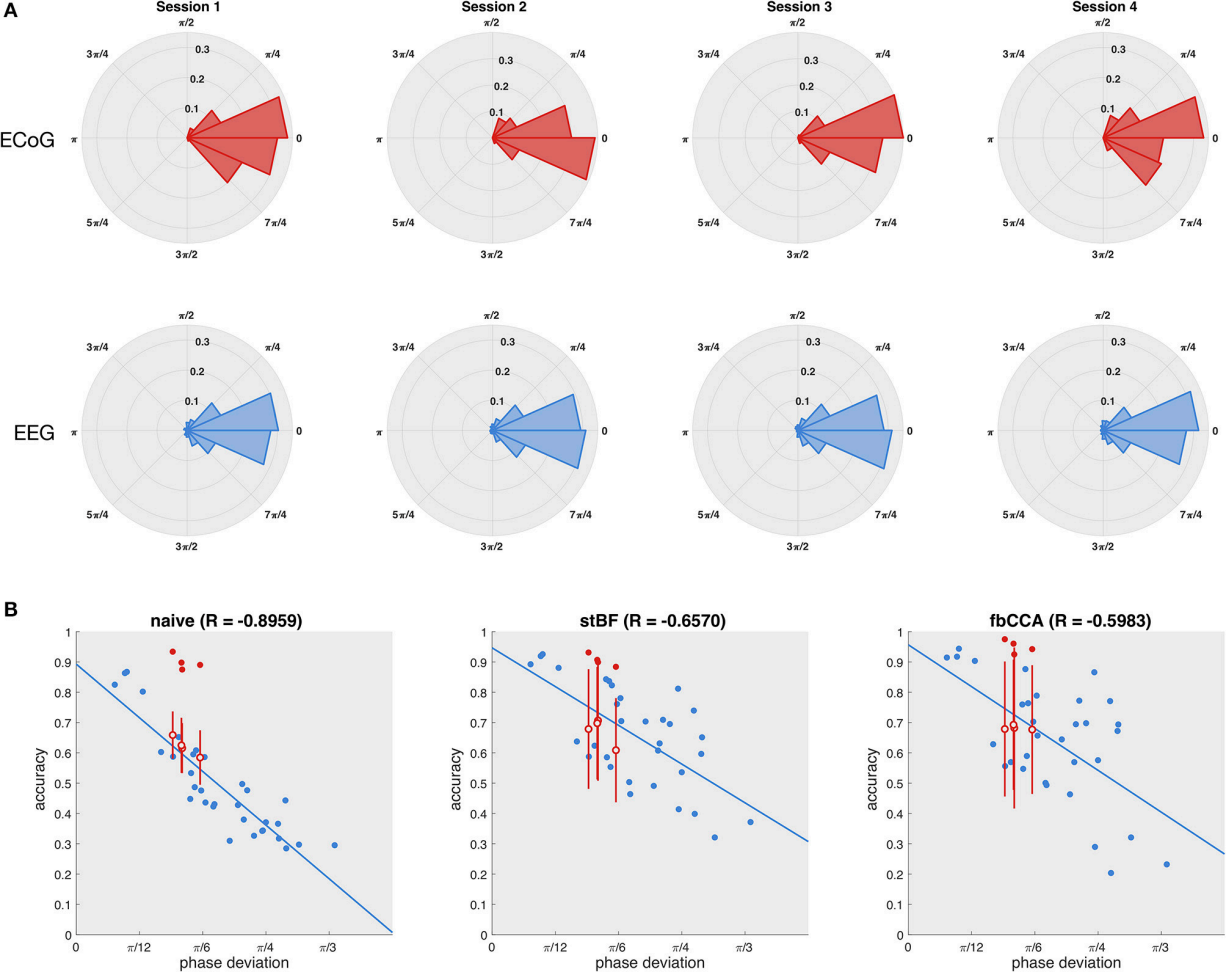
Estimation of the patient's scalp-EEG accuracy. **(A)** Distribution of the phase deviation at the stimulation frequency across trials of the ECoG (left column) and scalp-EEG (right column) for the four sessions (rows). Kuiper's test did not reveal any significant differences between the ECoG and scalp-EEG phase deviation distributions for any of the four sessions. **(B)** Regression of accuracy on phase deviation. Each dot represents one session of one subject. Blue dots indicate the control subjects, red filled dots indicate the patient, and red open dots indicate the statistically estimated scalp-EEG accuracy of the patient with the vertical red line the corresponding standard deviation for 100 imputations.

As the quality of the scalp-EEG of the patient was not sufficient, we statistically estimated his scalp-EEG decoding performance based on the measured phase of the ECoG signal. Figure 3A shows the distribution of the phase deviations across all targets for each session for both the ECoG (electrode 36) and EEG control group (electrode Oz). The distributions of ECoG and scalp-EEG are not statistically significant for all four sessions (*p* > 0.05, Kuiper's test), indicating that both ECoG and scalp-EEG experience similar phase deviations across trials, and that the higher classification accuracy of ECoG does not originate from more stable phase responses. Using the phase and accuracy measurements of the control group, the accuracy of the patient's scalp-EEG was statistically estimated using an imputation-analysis (Rubin, 1976; Carpenter and Kenward, 2012). Figure 3B shows a regression analysis of the accuracy (averaged over all stimulation lengths) with respect to the phase deviation based on the control group for all classifiers. Additionally, the accuracies and phase deviations of the ECoG signal at electrode 36 are added, as well as the estimations of the patient's scalp-EEG accuracy, given the observed ECoG phase deviations. While all three classifiers exhibit a decrease in accuracy for decreasing phase stability, the naive classifier is most strongly affected, probably as it does not adopt more advanced signal processing procedures. For all classifiers, the ECoG-based accuracies clearly outperform the scalp-EEG counterparts with similar phase deviations.

### 3.2. Multi-electrode performance

Since several EEG studies showed that the decoding accuracy of scalp-EEG improves when using a multi-electrode approach (Liu et al., 2013), we adopted a greedy forward electrode selection strategy to determine the electrode set for each classifier and subject individually.

**Figure 4:**
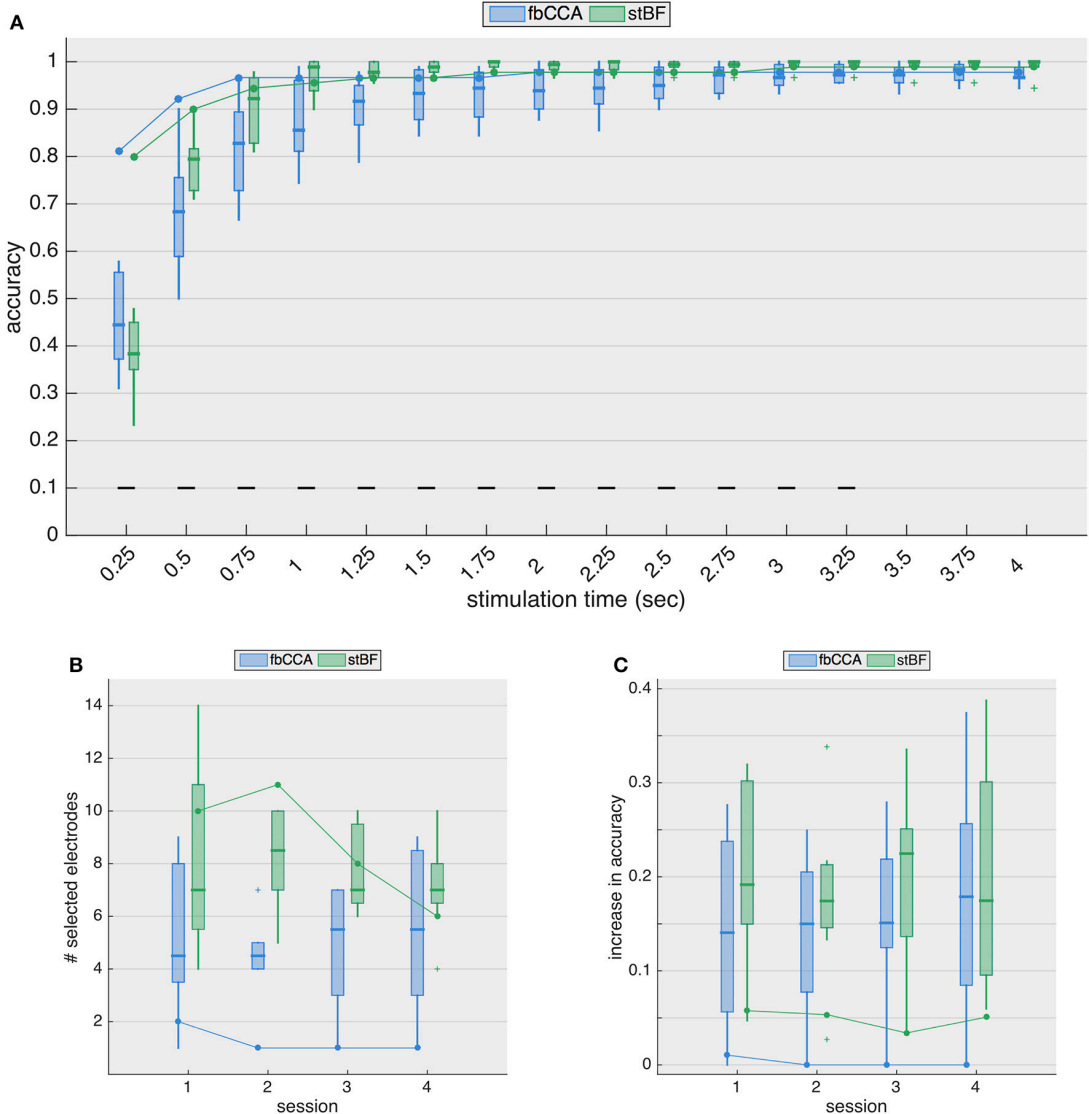
Multi-electrode performance. **(A)** Multi-electrode analysis of classification accuracies during the second session. **(B)** Number of electrodes selected by the greedy algorithm, for each session. Boxplots and solid line indicate results for EEG (control subjects) and ECoG (implanted patient), respectively. **(C)** Increase in accuracy (averaged over all stimulation lengths) for all sessions. Boxplots and solid line indicate results for EEG (control subjects) and ECoG (implanted patient), respectively.

Figure 4A shows the resulting classification accuracies for the second session. While the accuracies from ECoG largely remain the same as in the single-electrode case, for EEG, the accuracies are considerably improved. The stimulation length required to reach the 70% threshold now decreases to 0.5 and 0.75 s for stBF and fbCCA, respectively. The spatiotemporal beamformer significantly outperforms the filterbank CCA algorithm for signal lengths from 0.5 to 3.25 s, but significantly underperforms for the shortest stimulation length, albeit that the accuracies of both classifiers are not satisfactory (i.e., below 70%).

Figure 4B shows the number of electrodes selected for each session for both classifiers, and Figure 4C shows the general increase in accuracy (i.e., averaged accuracy across all stimulation lengths) for the four sessions. While the accuracies with EEG increase considerably (median improvement between 14 and 23%), those with ECoG increase only marginally (~ 5% for the stBF and ≤ 1% for fbCCA). For sessions 2 to 4, greedy electrode selection did not come up with additional electrodes for the fbCCA method (Figure 4B), resulting in identical accuracies as for the single-electrode case. Unlike for the fbCCA classifier, between 6 and 11 electrodes were included for the stBF in the ECoG modality, but this led to only minor improvements in decoding accuracy (Figure 4C).

## 4. Discussion

We investigated the accuracy with which SSVEP-selectable targets can be decoded from ECoG recordings, obtained from a subdural electrode grid spanning the entire right occipital cortex, when using classifiers that were originally designed for scalp-EEG recordings. We considered two state-of-the-art classifiers and one naive approach, based on spectral analysis and phase detection, and assessed their accuracies for both ECoG and scalp-EEG recordings in terms of stimulation length in single- and multi-electrode settings.

### 4.1. High-quality ECoG signals allow for accurate single-electrode SSVEP decoding

**Figure 5:**
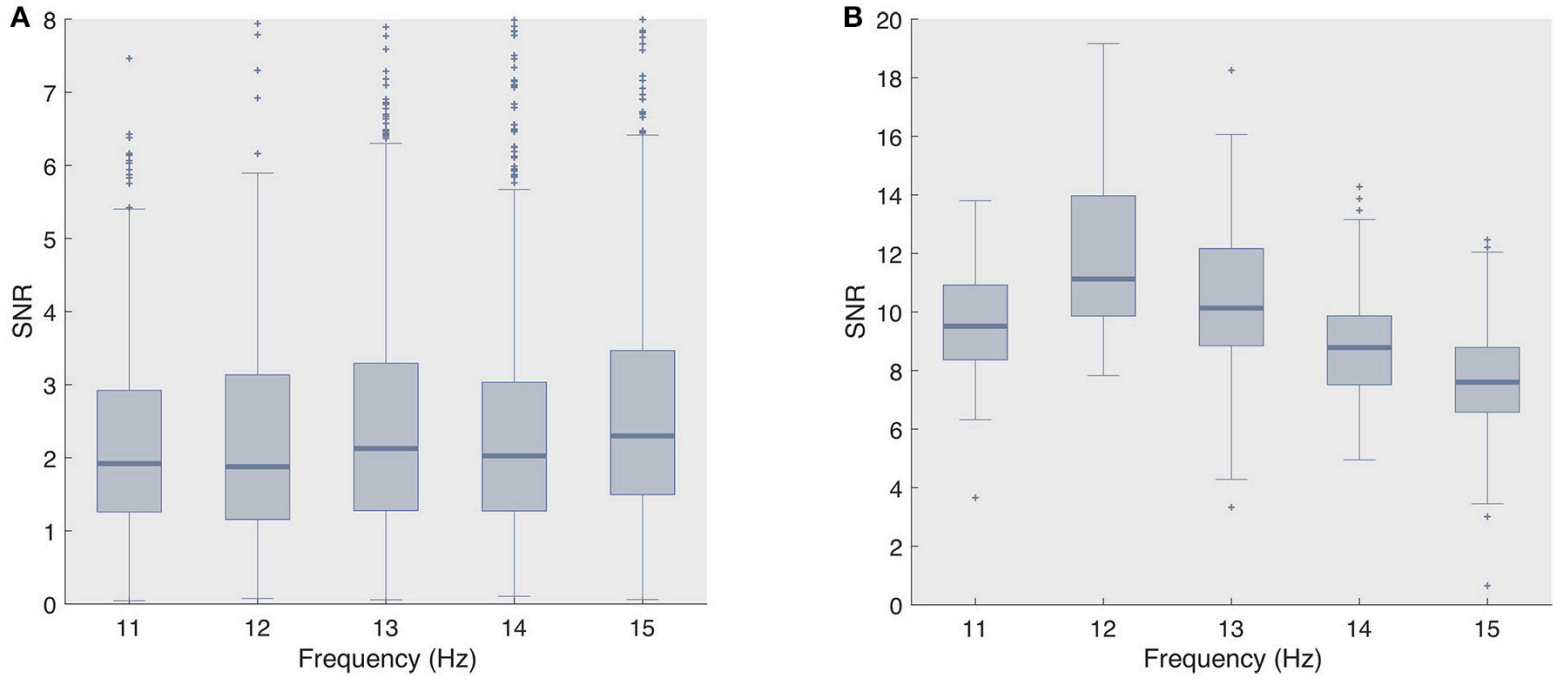
Signal-to-noise ratio's for all considered frequencies **(A)** scalp-recorded EEG and **(B)** ECoG.

The superior decoding accuracy of ECoG was most striking for the single-electrode analysis, as even the naive classifier surpassed 90% classification accuracy for stimulation lengths as short as 0.75 s. However, when adopting even shorter stimulation length (≤ 0.5 s), both state-of-the-art classifiers, and fbCCA in particular, yielded noticeably higher accuracies compared to the naive classifier, e.g., for the second session, we obtained 92.2 and 85.6% for fbCCA and stBF, respectively, compared to 72.2% for the naive classifier at a stimulation length of 0.5 s. In comparison, when using scalp-EEG, both state-of-the-art classifiers outperformed the naive classifier for all stimulation lengths, but required much longer stimulation times (more than 3 s) to reach 90% accuracy. The fact that the naive classifier attains high accuracy levels on ECoG supports the claim that subdural signals are of better quality. Figure 5 confirms this as the single-trial signal-to-noise ratio (SNR) of the subdural signal (at electrode 36) is considerably higher than that of its scalp-recorded counterpart (at electrode Oz).

In order not to interfere with the patient's clinical workup, we were not able to obtain simultaneous scalp-EEG from the patient. However, even in case simultaneous scalp-EEG was obtained, it is unclear whether the decoding accuracy would be useful for comparison, as several studies have presented initial evidence that the implanted silastic layer containing the subdural electrodes considerably attenuates the scalp-recorded potentials (Tao et al., 2005; Lanfer et al., 2013; Ramantani et al., 2014): a 2-3 times attenuation for a 4 × 8 grid and up to 8 times for an 8 × 8 grid (von Ellenrieder et al., 2014). As an alternative, we estimated the patient's scalp-EEG decoding accuracy from the ECoG phase variability using a data imputation exercise based on the fact that volume conduction affects the EEG signal's amplitude, not its phase. In the same vein, the head-models used in EEG source localization studies are resistive and therefore do not account for phase information (Sarvas, 1987; Hallez et al., 2007; Wolters and de Munck, 2007).

The high regression value for the naive classifier with respect to the scalp-EEG phase deviation (Figure 3) indicates that this classifier is mainly influenced by the consistency of the phase responses. However, despite that the variability of the subdural phase responses are not significantly different to those of the control group, the subdural decoding accuracy is considerably higher. This discrepancy in accuracy also shows up in the imputation analysis (Figure 3). We suggest that the higher decoding accuracy is due to the higher SNR of ECoG signals (Figure 5).

As for the naive classifier, the decoding accuracies of stBF and fbCCA are reduced when the phase deviation increases (Figure 3B). However, as the latter are more advanced classifiers, their regression coefficients are lower, indicating they are able to partially account for the trial-by-trial phase variability.

### 4.2. ECoG-based SSVEP decoding does not benefit from a multi-electrode approach

Assuming that information from other channels improves the classification accuracy, many researchers (for review Liu et al., 2013) developed spatial filtering and multi-electrode decoding strategies, including the ones considered in this study. When adopting a multi-electrode approach, the scalp-EEG decoding accuracies improve considerably for both classifiers, with an increased accuracy (averaged over all stimulation lengths) between 13 and 23%, depending on the considered session. In contrast, for ECoG, the decoding accuracy increased only marginally (~ 5%) with stBF, and even less for fbCCA, for which the greedy electrode selection did not include any additional electrode, as the accuracy could not be improved (with exception of the first session where 1 additional electrode was included). Overall, when opting for a multi-electrode approach, the accuracies obtained from ECoG and EEG were similar as soon as the stimulation length exceeded 1 s, demonstrating the effectiveness of current state-of-the-art SSVEP decoding algorithms.

In our previous work (Wittevrongel et al., 2018), we showed that the representation of the foveal flickering was highly localized at the posterior part of the primary visual cortex (electrodes 35 and 36), with little variation across the stimulation frequencies used. Also in the single-electrode analysis (Figure 2), the highest accuracies were obtained with the electrodes covering the posterior part of the primary visual cortex. The inclusion of additional subdural electrodes in the analysis did not improve decoding accuracy, indicating that the electrodes over the posterior part of the primary visual cortex carry the information needed for target decoding, and that there is little overlap with the information provided by the surrounding electrodes. This is in line with the literature that, compared to scalp-recorded potentials, cortical activations (both induced and noise) are considerably more localized (Buzsáki et al., 2012). As volume conduction attenuates and disperses the cortical activity over a larger scalp area, multiple scalp-electrodes carry information from the activity-of-interest. A multi-electrode analysis is therefore beneficial for decoding SSVEPs from the scalp, as it includes multiple weaker sources of information about the activity-of-interest.

### 4.3. Comparison with other visual BCI paradigms

ECoG has been hailed for BCI purposes for its long-term stability, superior signal quality and permanent availability of the interface (no electrode mounting required) (Wang et al., 2013; Vansteensel et al., 2016), but the reported applications rely on multiple electrodes, implanted as grids or strips, requiring a major surgical intervention. For instance, when pursuing a motor imagery application, one typically relies on a multi-electrode implants covering over the sensorimotor cortex to decode actions of different limbs, the activity of which is highly localized (Miller et al., 2010). Visual Event-Related Potential (ERP) paradigms have shown high accuracies in multi-electrode ECoG settings, but the accuracy considerably drops when adopting a single-electrode analysis (100% with 4 electrodes and 3 stimulus repetitions compared to 78% with a single electrode; Brunner et al., 2011). As to the cVEP paradigm, a multi-electrode analysis using CCA as spatial filter showed an accuracy of 86.8% with a stimulation time of 1.05 s (Kapeller et al., 2013). Compared to the paradigms mentioned above, as we have shown in this contribution, SSVEP stands out due to its high single-electrode decoding accuracy with short stimulation times (96.7% with a stimulation time of only 0.75 s). This could even be reached by using naive classifier based on the Fourier transform, without tailoring the frequency or phase characteristics to the subject. If the stimulus characteristics were optimized, the decoding performance could likely be further increased (Chen et al., 2015b).

### 4.4. Practical considerations and future work

Another important point in the comparison of scalp-EEG and ECoG accuracies is the conditions in which the recording was performed. Despite the impressive stimulation length/accuracy ratio we obtained with ECoG, it is worth mentioning that in most sessions the patient was unable to reach maximal accuracy (i.e., 100%), even with longer stimulation lengths. While some EEG subjects did reach maximal accuracy, it should be noted that our control subjects performed the experiment in a dedicated experimental room without distractions (sound-attenuated, air-conditioned, black walls), while the patient performed the experiment in a hospital room where many distractions were present (e.g., the experiment was interrupted several times to ensure normal continued medical treatment, surrounding relatives, and so on). Despite these suboptimal recording conditions, our results clearly demonstrate the superiority of ECoG compared to scalp-EEG for the SSVEP paradigm, both in terms of number of electrodes and stimulation lengths.

While the patient did not have a seizure during the experimental session, interictal activity might influence the recorded signals. However, the clinical functional mapping showed that none of the electrodes over V1 overlapped with the clinically determined ictal onset zone, which was located lateral from V2, indicating that the primary visual cortex of this patient can be considered healthy, normal tissue. The fact that the variability in the measured phase responses of ECoG and scalp-EEG was not significantly different and that the imputation analysis returned accuracies in the expected range provide further confidence that our results would generalize to other subjects and that there was no influence of the epileptic tissue on our results.

Given these encouraging findings, although based on offline analysis, we believe that our results could also pertain to an online implementation: the used state-of-the-art decoding algorithms have already been shown to also yield superior online decoding accuracy when based on scalp-EEG (Chen et al., 2015b; Wittevrongel and Van Hulle, 2016c).

### 4.5. Limitations

For this study, we recorded one subject implanted with a subdural grid over the occipital cortex. While it is generally unclear whether or not the results from a single subject generalize to the population, the current study provided an unique opportunity to study the cortical activations of the widely adopted SSVEP paradigm. As mentioned before, the prevalence of occipital lobe epilepsy is low. Only 5–10% of epilepsy cases have an epileptic generator in the occipital cortex (Sveinbjornsdottir and Duncan, 1993). Of this small group, only cases of intractable (and operable) epilepsy are candidate for intracranial implantation. Furthermore, one has to exclude photosensitive epilepsy patients, as SSVEPs are elicited by periodically flickering stimulation. Finally, as an alternative to electrode implantation, more and more surgeons currently prefer awake surgery during which they can perform functional mapping, thereby removing the need for electrode implantation. Given all these restrictions, it is very rare to study cortical activations in response to SSVEP stimulation *in-vivo* in a human subject, even more when the primary visual cortex is completely covered, including the interhemispheric fissure.

Unfortunately, as the hospital's EEG system was not set up for communicating real-time recordings to an external system, a closed-loop SSVEP experiment could not be performed.

## 5. Conclusion

The recording of brain signals directly from the cortical surface using electrocorticography (ECoG) has great benefits for BCI. However, the most performant visual BCI paradigm, based on the SSVEP, has never been assessed with ECoG, primarily due to the highly exceptional occurrence of subdural electrode implantation over the occipital cortex. In this work, we quantify the superior SSVEP decoding accuracies of ECoG compared to those of a control group with scalp-recorded electroencephalography (EEG), and show that, unlike with scalp-EEG, ECoG does not benefit from a multi-electrode analysis.

## Data availability statement

The datasets for this manuscript are not publicly available as it contains sensitive information about the patient, and therefore cannot be transferred using online repositories.

## Author contributions

EC, LD, AM, PB, and DV recruited the patient. BW and EK designed and implemented the experiment. BW, EK, LD, and EC collected the data. BW performed the analysis. All authors wrote and reviewed the manuscript.

### Conflict of interest statement

The authors declare that the research was conducted in the absence of any commercial or financial relationships that could be construed as a potential conflict of interest.

## References

[B1] Abu-Alqumsan M. Peer A. (2016). Advancing the detection of steady-state visual evoked potentials in brain–computer interfaces. J. Neural Eng. 13:036005. 10.1088/1741-2560/13/3/036005 27064728

[B2] Allison B. Luth T. Valbuena D. Teymourian A. Volosyak I. Graser A. (2010). BCI demographics: How many (and what kinds of) people can use an SSVEP BCI? IEEE Trans. Neural Syst. Rehabil. Eng. 18, 107–116. 10.1109/TNSRE.2009.2039495 20083463

[B3] Arvaneh M. Guan C. Ang K. K. Quek C. (2011). Optimizing the channel selection and classification accuracy in EEG-based BCI. IEEE Trans. Biomed. Eng. 58, 1865–1873. 10.1109/TBME.2011.2131142 21427014

[B4] Ball T. Kern M. Mutschler I. Aertsen A. Schulze-Bonhage A. (2009). Signal quality of simultaneously recorded invasive and non-invasive EEG. Neuroimage 46, 708–716. 10.1016/j.neuroimage.2009.02.028 19264143

[B5] Barachant A. Bonnet S. (2011). Channel selection procedure using riemannian distance for bci applications, in 2011 5th International IEEE/EMBS Conference on Neural Engineering (NER) (Cancun: IEEE), 348–351.

[B6] Berens P. (2009). Circstat: a matlab toolbox for circular statistics. J. Stat. Softw. 31, 1–21. 10.18637/jss.v031.i10

[B7] Bin G. Gao X. Yan Z. Hong B. Gao S. (2009). An online multi-channel SSVEP-based brain–computer interface using a canonical correlation analysis method. J. Neural Eng. 6:046002. 10.1088/1741-2560/6/4/046002 19494422

[B8] Brainard D. H. Vision S. (1997). The psychophysics toolbox. Spatial Vis. 10, 433–436. 10.1163/156856897X00357 9176952

[B9] Brunner P. Ritaccio A. L. Emrich J. F. Bischof H. Schalk G. (2011). Rapid communication with a “p300” matrix speller using electrocorticographic signals (ECoG). Front. Neurosci. 5:5. 10.3389/fnins.2011.00005 21369351 PMC3037528

[B10] Buzsáki G. Anastassiou C. A. Koch C. (2012). The origin of extracellular fields and currents—EEG, ECoG, LFP and spikes. Nat. Rev. Neurosci. 13, 407–420. 10.1038/nrn3241 22595786 PMC4907333

[B11] Cao T. Wan F. Mak P. U. Mak P.-I. Vai M. I. Hu Y. (2012). Flashing color on the performance of ssvep-based brain-computer interfaces, in Engineering in Medicine and Biology Society (EMBC), 2012 Annual International Conference of the IEEE (San Diego, CA: IEEE), 1819–1822. 10.1109/EMBC.2012.6346304 23366265

[B12] Carpenter J. Kenward M. (2012). Multiple Imputation and Its Application. Chichester: John Wiley & Sons.

[B13] Chao Z. C. Nagasaka Y. Fujii N. (2010). Long-term asynchronous decoding of arm motion using electrocorticographic signals in monkeys. Front. Neuroeng. 3:3. 10.3389/fneng.2010.00003 20407639 PMC2856632

[B14] Chen X. Wang Y. Gao S. Jung T.-P. Gao X. (2015a). Filter bank canonical correlation analysis for implementing a high-speed SSVEP-based brain–computer interface. J. Neural Eng. 12:046008. 10.1088/1741-2560/12/4/046008 26035476

[B15] Chen X. Wang Y. Nakanishi M. Gao X. Jung T.-P. Gao S. (2015b). High-speed spelling with a noninvasive brain–computer interface. Proc. Natl. Acad. Sci. U.S.A. 112, E6058–E6067. 10.1073/pnas.1508080112 26483479 PMC4640776

[B16] Chen X. Wang Y. Nakanishi M. Jung T.-P. Gao X. (2014). Hybrid frequency and phase coding for a high-speed SSVEP-based BCI speller. in Engineering in Medicine and Biology Society (EMBC), 2014 36th Annual International Conference of the IEEE (IEEE), 3993–3996. 10.1109/EMBC.2014.6944499 25570867

[B17] Collinger J. L. Wodlinger B. Downey J. E. Wang W. Tyler-Kabara E. C. Weber D. J.. (2013). High-performance neuroprosthetic control by an individual with tetraplegia. Lancet 381, 557–564. 10.1016/S0140-6736(12)61816-9 23253623 PMC3641862

[B18] Combaz A. Chatelle C. Robben A. Vanhoof G. Goeleven A. Thijs V.. (2013). A comparison of two spelling brain-computer interfaces based on visual P3 and SSVEP in locked-in syndrome. PLoS ONE 8:e73691. 10.1371/journal.pone.0073691 24086289 PMC3783473

[B19] Croft R. J. Barry R. J. (2000). Removal of ocular artifact from the EEG: a review. Clin. Neurophysiol. 30, 5–19. 10.1016/S0987-7053(00)00055-1 10740792

[B20] Fischl B. (2012). Freesurfer. Neuroimage 62, 774–781. 10.1016/j.neuroimage.2012.01.021 22248573 PMC3685476

[B21] Guyon I. Elisseeff A. (2003). An introduction to variable and feature selection. J. Mach. Learn. Res. 3, 1157–1182.

[B22] Hallez H. Vanrumste B. Grech R. Muscat J. De Clercq W. Vergult A.. (2007). Review on solving the forward problem in EEG source analysis. J. Neuroeng. Rehabil. 4:46. 10.1186/1743-0003-4-46 18053144 PMC2234413

[B23] Härdle W. K. Simar L. (2015). Canonical correlation analysis, in Applied Multivariate Statistical Analysis, (Berlin; Heidelberg: Springer), 443–454.

[B24] Hochberg L. R. Serruya M. D. Friehs G. M. Mukand J. A. Saleh M. Caplan A. H.. (2006). Neuronal ensemble control of prosthetic devices by a human with tetraplegia. Nature 442:164. 10.1038/nature04970 16838014

[B25] Jia C. Gao X. Hong B. Gao S. (2011). Frequency and phase mixed coding in SSVEP-based brain–computer interface. IEEE Trans. Biomed. Eng. 58, 200–206. 10.1109/TBME.2010.2068571 20729160

[B26] Jiang T. Jiang T. Wang T. Mei S. Liu Q. Li Y.. (2017). Characterization and decoding the spatial patterns of hand extension/flexion using high-density ECoG. IEEE Trans. Neural Syst. Rehabil. Eng. 25, 370–379. 10.1109/TNSRE.2016.2647255 28060708

[B27] Kaiser P. K. (1984). Physiological response to color: a critical review. Color Res. Appl. 9, 29–36. 10.1002/col.5080090106

[B28] Kamp A. Sem-Jacobsen C. Leeuwen W. van der Tweel L. (1960). Cortical responses to modulated light in the human subject. Acta Physiol. 48, 1–12. 10.1111/j.1748-1716.1960.tb01840.x 14404245

[B29] Kapeller C. Kamada K. Ogawa H. Prueckl R. Scharinger J. Guger C. (2013). An electrocorticographic BCI using code-based VEP for control in video applications: a single-subject study. Front. Syst. Neurosci. 8:139. 10.3389/fnsys.2014.00139 25147509 PMC4124519

[B30] Kleiner M. Brainard D. Pelli D. Ingling A. Murray R. Broussard C. (2007). What's new in psychtoolbox-3. Perception 36:1.

[B31] Korik A. Sosnik R. Siddique N. Coyle D. (2018). Decoding imagined 3D hand movement trajectories from EEG: evidence to support the use of mu, beta, and low gamma oscillations. Front. Neurosci. 12:130. 10.3389/fnins.2018.00130 29615848 PMC5869206

[B32] Krolak-Salmon P. Hénaff M.-A. Tallon-Baudry C. Yvert B. Guénot M. Vighetto A.. (2003). Human lateral geniculate nucleus and visual cortex respond to screen flicker. Ann. Neurol. 53, 73–80. 10.1002/ana.10403 12509850

[B33] Krusienski D. J. Shih J. J. (2011). Spectral components of the p300 speller response in electrocorticography, in 2011 5th International IEEE/EMBS Conference on Neural Engineering (NER) (Cancun: IEEE), 282–285.

[B34] Kübler A. Birbaumer N. (2008). Brain-computer interfaces and communication in paralysis: extinction of goal directed thinking in completely paralysed patients? Clin. Neurophysiol. 119, 2658–2666. 10.1016/j.clinph.2008.06.019 18824406 PMC2644824

[B35] Kübler A. Neumann N. Wilhelm B. Hinterberger T. Birbaumer N. (2004). Predictability of brain-computer communication. J. Psychophysiol. 18, 121–129. 10.1027/0269-8803.18.23.121

[B36] Lacko D. Vleugels J. Fransen E. Huysmans T. De Bruyne G. Van Hulle M. M.. (2017). Ergonomic design of an EEG headset using 3D anthropometry. Appl. Ergon. 58, 128–136. 10.1016/j.apergo.2016.06.002 27633205

[B37] Lal T. N. Schroder M. Hinterberger T. Weston J. Bogdan M. Birbaumer N.. (2004). Support vector channel selection in BCI. IEEE Trans. Biomed. Eng. 51, 1003–1010. 10.1109/TBME.2004.827827 15188871

[B38] Lanfer B. Röer C. Scherg M. Rampp S. Kellinghaus C. Wolters C. (2013). Influence of a silastic ECoG grid on EEG/ECoG based source analysis. Brain Topogr. 26, 212–228. 10.1007/s10548-012-0251-0 22941500

[B39] Lee P.-L. Sie J.-J. Liu Y.-J. Wu C.-H. Lee M.-H. Shu C.-H.. (2010). An SSVEP-actuated brain computer interface using phase-tagged flickering sequences: a cursor system. Anna. Biomed. Eng. 38, 2383–2397. 10.1007/s10439-010-9964-y 20177780

[B40] Leuthardt E. C. Schalk G. Wolpaw J. R. Ojemann J. G. Moran D. W. (2004). A brain–computer interface using electrocorticographic signals in humans. J. Neural Eng. 1, 63–71. 10.1088/1741-2560/1/2/001 15876624

[B41] Lin Z. Zhang C. Wu W. Gao X. (2006). Frequency recognition based on canonical correlation analysis for SSVEP-based BCIs. IEEE Trans. Biomed. Eng. 53, 2610–2614. 10.1109/TBME.2006.886577 17152442

[B42] Liu Q. Chen K. Ai Q. Xie S. Q. (2013). Review: recent development of signal processing algorithms for SSVEP-based brain computer interfaces. J. Med. Biol. Eng. 34, 299–309.

[B43] Lopez-Gordo M. Prieto A. Pelayo F. Morillas C. (2010). Use of phase in brain–computer interfaces based on steady-state visual evoked potentials. Neural Process. Lett. 32, 1–9. 10.1007/s11063-010-9139-8

[B44] Lotte F. Bougrain L. Cichocki A. Clerc M. Congedo M. Rakotomamonjy A.. (2018). A review of classification algorithms for EEG-based brain–computer interfaces: a 10 year update. J. Neural Eng. 15:031005. 10.1088/1741-2552/aab2f2 29488902

[B45] Luo A. Sullivan T. J. (2010). A user-friendly SSVEP-based brain–computer interface using a time-domain classifier. J. Neural Eng. 7:026010. 10.1088/1741-2560/7/2/026010 20332551

[B46] Lv J. Liu M. (2008). Common spatial pattern and particle swarm optimization for channel selection in BCI, in 3rd International Conference on Innovative Computing Information and Control, 2008, ICICIC'08 (Dalian: IEEE), 457–457.

[B47] Manyakov N. V. Chumerin N. Combaz A. Robben A. Van Hulle M. M. (2010). Decoding SSVEP responses using time domain classification, in IJCCI (Valencia: ICFC–ICNC), 376–380.

[B48] Manyakov N. V. Chumerin N. Robben A. Combaz A. van Vliet M. Van Hulle M. M. (2013). Sampled sinusoidal stimulation profile and multichannel fuzzy logic classification for monitor-based phase-coded SSVEP brain–computer interfacing. J. Neural Eng. 10:036011. 10.1088/1741-2560/10/3/036011 23594762

[B49] Manyakov N. V. Chumerin N. Van Hulle M. M. (2012). Multichannel decoding for phase-coded SSVEP brain–computer interface. Int. J. Neural Syst. 22:1250022. 10.1142/S0129065712500220 22963395

[B50] Middendorf M. McMillan G. Calhoun G. Jones K. S. (2000). Brain-computer interfaces based on the steady-state visual-evoked response. IEEE Trans. Rehabil. Eng. 8, 211–214. 10.1109/86.847819 10896190

[B51] Miller K. J. Schalk G. Fetz E. E. Den Nijs M. Ojemann J. G. Rao R. P. (2010). Cortical activity during motor execution, motor imagery, and imagery-based online feedback. Proc. Natl. Acad. Sci. U.S.A. 107, 4430–4435. 10.1073/pnas.0913697107 20160084 PMC2840149

[B52] Miller K. J. Sorensen L. B. Ojemann J. G. Den Nijs M. (2009b). Power-law scaling in the brain surface electric potential. PLoS Comput. Biol. 5:e1000609. 10.1371/journal.pcbi.1000609 20019800 PMC2787015

[B53] Miller K. J. Zanos S. Fetz E. Den Nijs M. Ojemann J. (2009a). Decoupling the cortical power spectrum reveals real-time representation of individual finger movements in humans. J. Neurosci. 29, 3132–3137. 10.1523/JNEUROSCI.5506-08.2009 19279250 PMC6666461

[B54] Nakanishi M. Wang Y. Wang Y.-T. Mitsukura Y. Jung T.-P. (2014). A high-speed brain speller using steady-state visual evoked potentials. Int. J. Neural Syst. 24:1450019. 10.1142/S0129065714500191 25081427

[B55] Nicolas-Alonso L. F. Gomez-Gil J. (2012). Brain computer interfaces, a review. Sensors 12, 1211–1279. 10.3390/s120201211 22438708 PMC3304110

[B56] Onose G. Grozea C. Anghelescu A. Daia C. Sinescu C. Ciurea A.. (2012). On the feasibility of using motor imagery EEG-based brain–computer interface in chronic tetraplegics for assistive robotic arm control: a clinical test and long-term post-trial follow-up. Spinal Cord 50:599. 10.1038/sc.2012.14 22410845

[B57] Pan J. Gao X. Duan F. Yan Z. Gao S. (2011). Enhancing the classification accuracy of steady-state visual evoked potential-based brain–computer interfaces using phase constrained canonical correlation analysis. J. Neural Eng. 8:036027. 10.1088/1741-2560/8/3/036027 21566275

[B58] Pelli D. G. (1997). The videotoolbox software for visual psychophysics: transforming numbers into movies. Spatial Vis. 10, 437–442. 10.1163/156856897X00366 9176953

[B59] Ramantani G. Dümpelmann M. Koessler L. Brandt A. Cosandier-Rimélé D. Zentner J.. (2014). Simultaneous subdural and scalp EEG correlates of frontal lobe epileptic sources. Epilepsia 55, 278–288. 10.1111/epi.12512 24417775

[B60] Regan D. (1979). Electrical responses evoked from the human brain. Sci. Am. 241, 134–146. 10.1038/scientificamerican1279-134 504980

[B61] Regan D. (1989). Human Brain Electrophysiology: Evoked Potentials and Evoked Magnetic Fields in Science and Medicine. Elsevier.

[B62] Ritaccio A. Matsumoto R. Morrell M. Kamada K. Koubeissi M. Poeppel D.. (2015). Proceedings of the seventh international workshop on advances in electrocorticography. Epilepsy Behav. 51, 312–320. 10.1016/j.yebeh.2015.08.002 26322594 PMC4593746

[B63] Rubin D. B. (1976). Inference and missing data. Biometrika 63, 581–592. 10.1093/biomet/63.3.581

[B64] Sarvas J. (1987). Basic mathematical and electromagnetic concepts of the biomagnetic inverse problem. Phys. Med. Biol. 32:11. 10.1088/0031-9155/32/1/004 3823129

[B65] Schalk G. (2010). Can electrocorticography (ECoG) support robust and powerful brain-computer interfaces? Front. Neuroeng. 3:9. 10.3389/fneng.2010.00009 20631853 PMC2903308

[B66] Schalk G. Leuthardt E. C. (2011). Brain-computer interfaces using electrocorticographic signals. IEEE Rev. Biomed. Eng. 4, 140–154. 10.1109/RBME.2011.2172408 22273796

[B67] Schröder M. Lal T. N. Hinterberger T. Bogdan M. Hill N. J. Birbaumer N. (2005). Robust EEG channel selection across subjects for brain-computer interfaces. EURASIP J. Appl. Signal Process. 2005, 3103–3112.

[B68] Shain W. Spataro L. Dilgen J. Haverstick K. Retterer S. Isaacson M.. (2003). Controlling cellular reactive responses around neural prosthetic devices using peripheral and local intervention strategies. IEEE Trans. Neural Syst. Rehabil. Eng. 11, 186–188. 10.1109/TNSRE.2003.814800 12899270

[B69] Speier W. Fried I. Pouratian N. (2013). Improved p300 speller performance using electrocorticography, spectral features, and natural language processing. Clin. Neurophysiol. 124, 1321–1328. 10.1016/j.clinph.2013.02.002 23465430 PMC3679217

[B70] Staba R. J. Wilson C. L. Bragin A. Fried I. Engel J. (2002). Quantitative analysis of high-frequency oscillations (80–500 hz) recorded in human epileptic hippocampus and entorhinal cortex. J. Neurophysiol. 88, 1743–1752. 10.1152/jn.2002.88.4.1743 12364503

[B71] Sveinbjornsdottir S. Duncan J. (1993). Parietal and occipital lobe epilepsy: a review. Epilepsia 34, 493–521. 10.1111/j.1528-1157.1993.tb02590.x 8504783

[B72] Tadel F. Baillet S. Mosher J. C. Pantazis D. Leahy R. M. (2011). Brainstorm: a user-friendly application for meg/eeg analysis. Comput. Intell. Neurosci. 2011:8. 10.1155/2011/879716 21584256 PMC3090754

[B73] Tao J. X. Ray A. Hawes-Ebersole S. Ebersole J. S. (2005). Intracranial EEG substrates of scalp EEG interictal spikes. Epilepsia 46, 669–676. 10.1111/j.1528-1167.2005.11404.x 15857432

[B74] Tello R. J. Müller S. M. T. Ferreira A. Bastos T. F. (2015). Comparison of the influence of stimuli color on steady-state visual evoked potentials. Res. Biomed. Eng. 31, 218–231. 10.1590/2446-4740.0739

[B75] Van Veen B. D. Buckley K. M. (1988). Beamforming: a versatile approach to spatial filtering. IEEE ASSP Mag. 5, 4–24. 10.1109/53.665

[B76] Van Veen B. D. Van Drongelen W. Yuchtman M. Suzuki A. (1997). Localization of brain electrical activity via linearly constrained minimum variance spatial filtering. IEEE Trans. Biomed. Eng. 44, 867–880. 10.1109/10.623056 9282479

[B77] van Vliet M. Chumerin N. De Deyne S. Wiersema J. Fias W. Storms G. Van Hulle M. (2015). Single-trial ERP component analysis using a spatio-temporal LCMV beamformer. IEEE Trans. Biomed. Eng. 63, 55–66. 10.1109/TBME.2015.2468588 26285053

[B78] Vansteensel M. J. Pels E. G. Bleichner M. G. Branco M. P. Denison T. Freudenburg Z. V. (2016). Fully implanted brain–computer interface in a locked-in patient with als. N. Engl. J. Med. 2016, 2060–2066. 10.1056/NEJMoa1608085 PMC5326682 27959736

[B79] Vetterli M. Herley C. (1992). Wavelets and filter banks: theory and design. IEEE Trans. Signal Process. 40, 2207–2232. 10.1109/78.157221

[B80] Vialatte F.-B. Maurice M. Dauwels J. Cichocki A. (2010). Steady-state visually evoked potentials: focus on essential paradigms and future perspectives. Prog. Neurobiol. 90, 418–438. 10.1016/j.pneurobio.2009.11.005 19963032

[B81] von Ellenrieder N. Beltrachini L. Muravchik C. H. Gotman J. (2014). Extent of cortical generators visible on the scalp: effect of a subdural grid. Neuroimage 101, 787–795. 10.1016/j.neuroimage.2014.08.009 25117602

[B82] Vu H. Koo B. Choi S. (2016). Frequency detection for SSVEP-based BCI using deep canonical correlation analysis, in 2016 IEEE International Conference on Systems, Man, and Cybernetics (SMC) (Budapest: IEEE), 001983–001987.

[B83] Walker J. S. (1988). Fourier Analysis. Cambridge, UK: Oxford University Press.

[B84] Wang W. Collinger J. L. Degenhart A. D. Tyler-Kabara E. C. Schwartz A. B. Moran D. W.. (2013). An electrocorticographic brain interface in an individual with tetraplegia. PLoS ONE 8:e55344. 10.1371/journal.pone.0055344 23405137 PMC3566209

[B85] Winawer J. Kay K. N. Foster B. L. Rauschecker A. M. Parvizi J. Wandell B. A. (2013). Asynchronous broadband signals are the principal source of the bold response in human visual cortex. Curr. Biol. 23, 1145–1153. 10.1016/j.cub.2013.05.001 23770184 PMC3710543

[B86] Wittevrongel B. Khachatryan E. Hnazaee M. F. Carrette E. De Taeye L. Meurs A.. (2018). Representation of steady-state visual evoked potentials elicited by luminance flicker in human occipital cortex: an electrocorticography study. Neuroimage 175, 315–326. 10.1016/j.neuroimage.2018.04.006 29630994

[B87] Wittevrongel B. Van Hulle M. M. (2016a). Faster p300 classifier training using spatiotemporal beamforming. Int. J. Neural Syst. 26:1650014. 10.1142/S0129065716500143 26971787

[B88] Wittevrongel B. Van Hulle M. M. (2016b). Frequency-and phase encoded SSVEP using spatiotemporal beamforming. PLoS ONE 11:e0159988. 10.1371/journal.pone.0159988 27486801 PMC4972379

[B89] Wittevrongel B. Van Hulle M. M. (2016c). Hierarchical online SSVEP spelling achieved with spatiotemporal beamforming, in Statistical Signal Processing Workshop (SSP), 2016 IEEE (Palma de Mallorca: IEEE), 1–5.

[B90] Wittevrongel B. Van Hulle M. M. (2017). Spatiotemporal beamforming: a transparent and unified decoding approach to synchronous visual brain-computer interfacing. Front. Neurosci. 11:630. 10.3389/fnins.2017.00630 29187809 PMC5695157

[B91] Wittevrongel B. Van Wolputte E. Van Hulle M. M. (2017). Code-modulated visual evoked potentials using fast stimulus presentation and spatiotemporal beamformer decoding. Sci. Rep. 7:15037. 10.1038/s41598-017-15373-x 29118386 PMC5678079

[B92] Wolters C. de Munck J. C. (2007). Volume conduction. Scholarpedia 2:1738 10.4249/scholarpedia.1738

[B93] Yin E. Zhou Z. Jiang J. Yu Y. Hu D. (2015). A dynamically optimized SSVEP brain-computer interface (BCI) speller. IEEE Trans. Biomed. Eng. 62, 1447–1456. 10.1109/TBME.2014.2320948 24801483

[B94] Zhang Y. Zhou G. Jin J. Wang X. Cichocki A. (2014). Frequency recognition in SSVEP-based BCI using multiset canonical correlation analysis. Int. J. Neural Syst. 24:1450013. 10.1142/S0129065714500130 24694168

[B95] Zhang Y. Zhou G. Zhao Q. Onishi A. Jin J. Wang X. Cichocki A. (2011). Multiway canonical correlation analysis for frequency components recognition in SSVEP-based BCIs, in Neural Information Processing (Shanghai: Springer), 287–295. 10.1007/978-3-642-24955-6_35

