# Supplementary material for: Decoding Steady-State Visual Evoked Potentials From Electrocorticography
Source: Front Neuroinform. 2018 Sep 26;12:65. doi: 10.3389/fninf.2018.00065 (PMC6168710; doi:10.3389/fninf.2018.00065)
Supplement: Supplementary file 1 [file Data_Sheet_1.PDF]

# *Supplementary Material*: Decoding Steady-State Visual Evoked Potentials from Electrocorticography

**Benjamin Wittevrongel, Elvira Khachatryan, Mansoureh Fahimi Hnazaee, Flavio Camarrone, Evelien Carrette, Leen De Taeye, Alfred Meurs, Paul Boon, Dirk Van Roost, Marc M Van Hulle**

*Correspondence:
Author Name: Benjamin Wittevrongel
benjamin.wittevrongel@kuleuven.be

## 1 SUPPLEMENTARY TABLES AND FIGURES

### 1.1 Figures

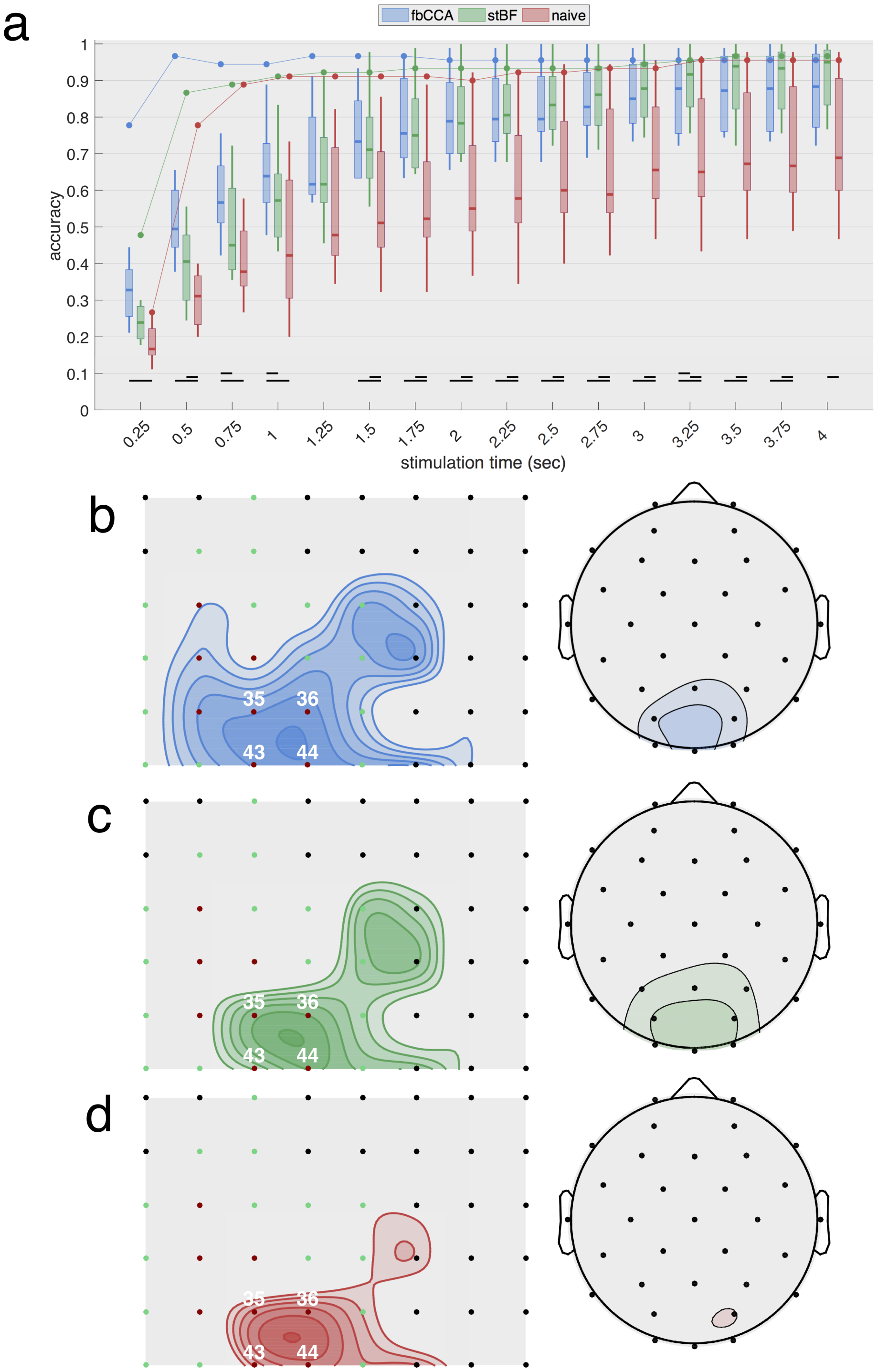


**Figure S1.** Single-electrode performance. (a) Best single-electrode accuracies obtained with the three classifiers plotted as a function of stimulation lengths during the first session. The full line indicates accuracies based on cortical recordings and the boxplots summarize accuracies based on scalp-EEG (control subjects). (b-d) Overview of accuracies (averaged across all stimulation lengths) for all cortical electrodes (left) and scalp channels (right) for (b) fbCCA, (c) stBF and (d) naive classifier. Subdural electrodes indicated in red and green indicate V1 and V2, respectively. Iso-accuracy lines on the subdural grids (left panels) start at 75% accuracy and increase in steps of 5% and on the scalp plots (right panels) start at 50% accuracy and increase in steps of 10%.

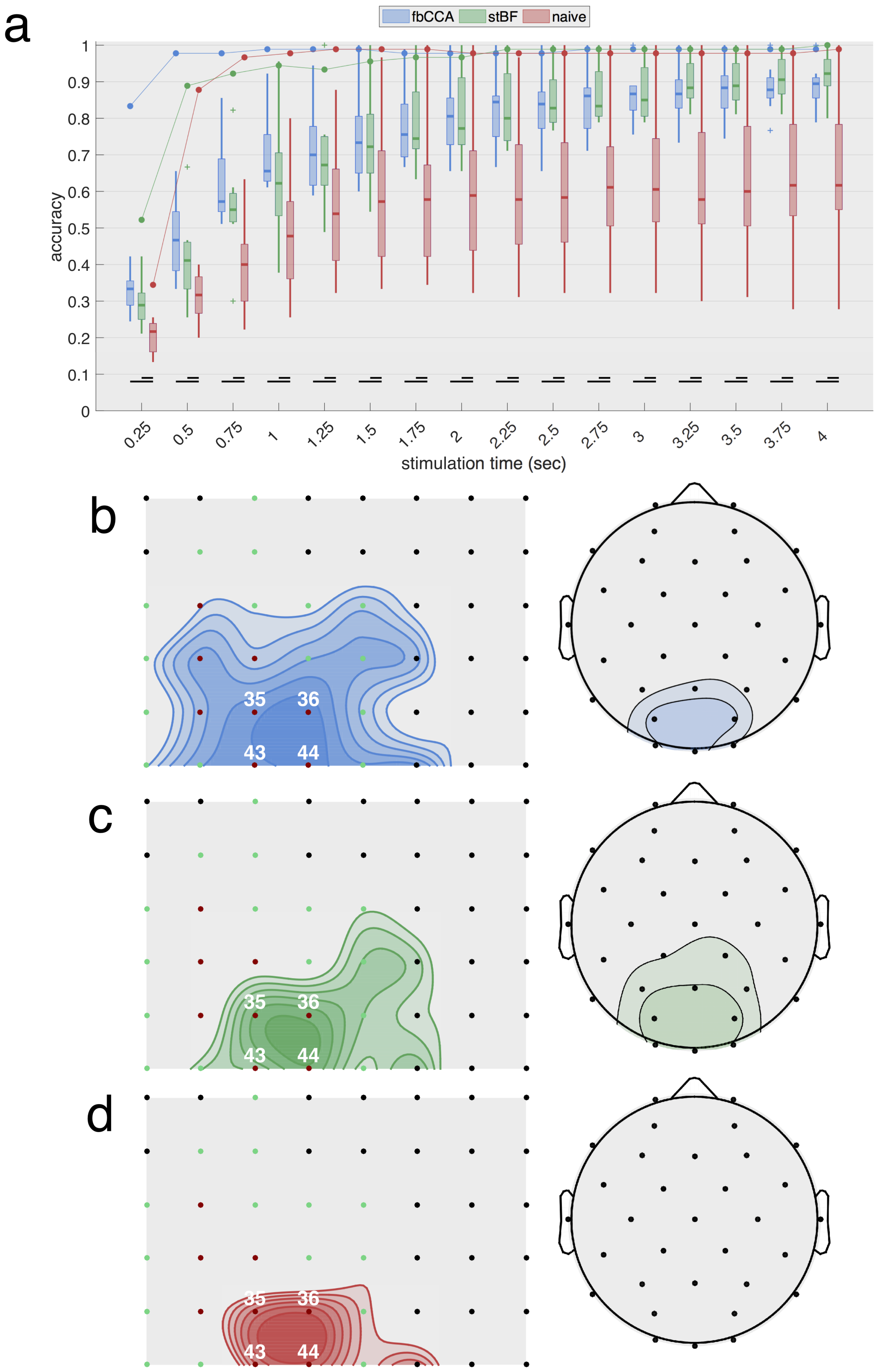


**Figure S2.** Accuracies of the best channel and overview of the accuracies of all channels for the third session. Same conventions as in Figure S1.

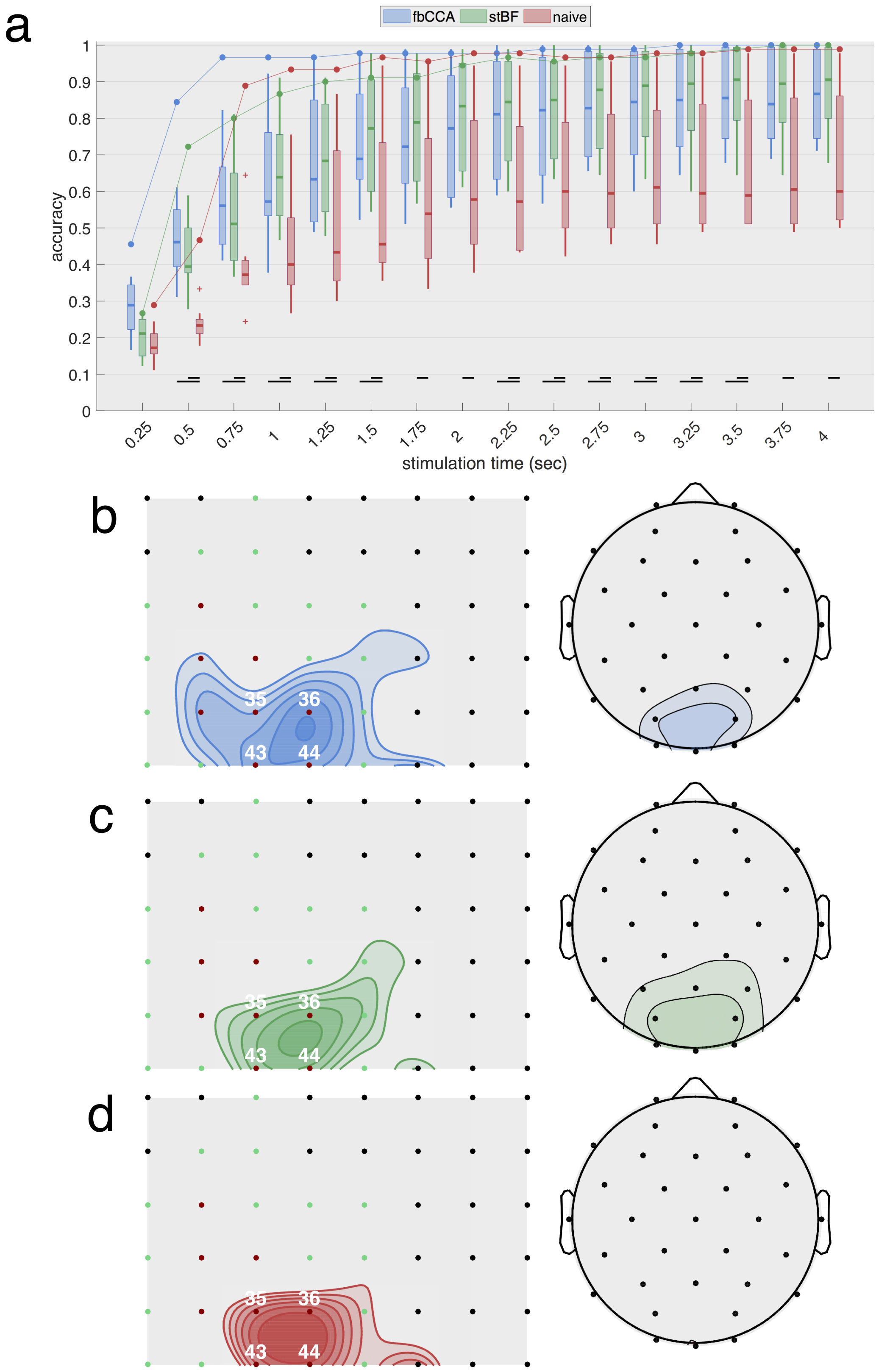


**Figure S3.** Accuracies of the best channel and overview of the accuracies of all channels for the fourth session. Same conventions as in Figure S1.

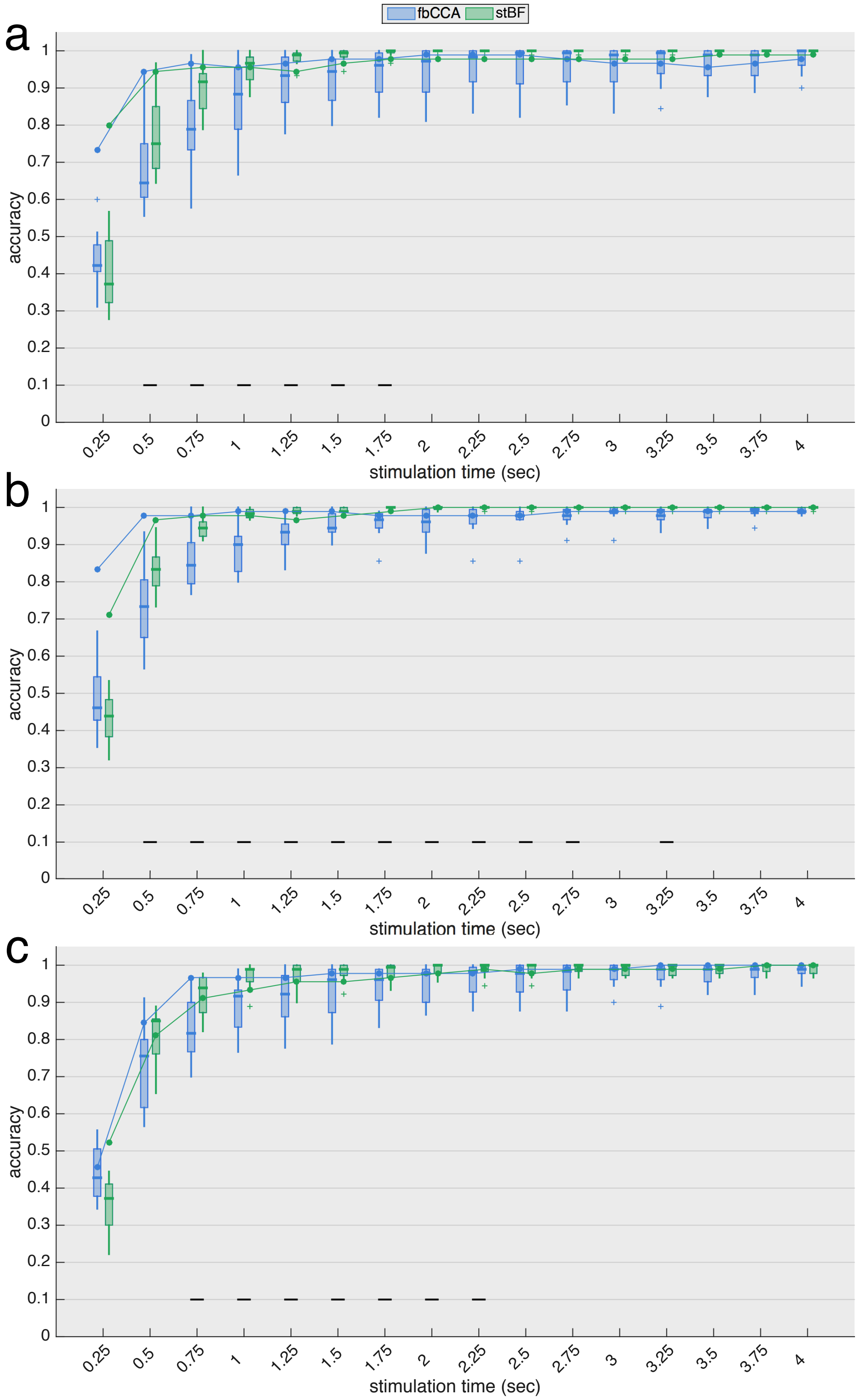


**Figure S4.** Accuracies of the multi-electrode analysis during (a) session 1, (b) session 3, and (c) session 4. The boxplots and full lines indicate the EEG subjects and ECoG patient, respectively.
